# Effects of different lifting strategies during resistance training on lower body function in untrained adult women: a comparison between 6-weeks of 10% velocity loss and standard resistance training

**DOI:** 10.3389/fspor.2025.1705675

**Published:** 2026-01-12

**Authors:** Matic Sašek, Hana Golob, Nejc Šarabon

**Affiliations:** 1 Faculty of Health Sciences, University of Primorska, Izola, Slovenia; 2 Andrej Marušič Institute, University of Primorska, Koper, Slovenia; 3 Laboratory for Motor Control and Motor Behavior, S2P, Science to Practice, Ltd, Kranj, Slovenia; 4 Ludwig Boltzmann Institute for Rehabilitation Research, Vienna, Austria

**Keywords:** adult women, dynapenia, muscle performance, power, powerpenia, resistance training, strength, velocity loss

## Abstract

**Introduction:**

This study investigated whether velocity-based resistance training provides additional benefits to lower limb performance compared to standard exercise execution.

**Methods:**

Twenty untrained adult women (37–55 years) were randomly assigned to two resistance training groups to perform resistance training with three sets of four lower body exercises per week for 6 weeks. The number of repetitions and lifting velocity differed between the groups. One group performed lower body exercises with maximal intent and a 10% velocity loss threshold termination (VB10%; *n* = 10), while the other group performed 10 repetitions at a standard 1:2 s concentric:eccentric tempo (STD; *n* = 10). The number of repetitions was recorded during the sessions. Before and after the intervention, power, muscular endurance and dynamic stability of the lower limbs were assessed using the mean propulsive velocity (MPV) and power (MPP) at 70% one-repetition maximum in the squat and deadlift, the Y-balance test (YBT) and the 30-second sit-to-stand test (STS), respectively. A two-way analysis of variance was used to assess the effects of time, group, and their interaction.

**Results:**

The difference between 10 repetitions in the STD and repetitions in the VB10% was assessed using a one-sample t-test. Both groups significantly improved MPP, MPV, YBT and STS [mean difference (MD) ≥5.4%; effect size (ES) ≥0.6]. Although 2.5–2.7 less repetitions were performed in VB10%, the improvements in MPP and MPV were slightly greater (ES ≥ 1.2 vs. ≥ 0.8). Conversely, STS and YBT improved more in STD (ES ≥ 0.4 vs. ≥ 1.0).

**Discussion:**

Regardless of the lifting method used, the training intervention improved lower limb power, muscular endurance and dynamic stability, indicating that resistance training is an effective strategy for enhancing these capacities in untrained adult women. Using 10% threshold may be a more time-efficient strategy for improving lower-limb power in this population and could represent a promising approach for mitigating early declines in power over time.

## 1 Introduction

The natural ageing process in women is associated with a progressive deterioration of neuromuscular function that begins in the third decade of life and accelerates after menopause due to endocrine changes, malnutrition, and reduced physical activity (1, 2). This deterioration is most evident in muscle mass, strength, and power of the lower body, which are essential for performing everyday tasks and maintaining health (3). When properly addressed, this decline in muscle function can be attenuated, contributing to improved functional capacity, quality of life, and physical and mental health outcomes (4, 5).

For this reason, resistance training has long been established as a key intervention to mitigate the age-related loss of muscle mass and strength, traditionally referred to as sarcopenia or dynapenia (6). However, recent evidence indicates that muscle power, defined as the product of force and movement velocity, declines earlier and more rapidly than muscle mass or strength and shows a stronger association with functional limitations in middle-aged women (7). This emerging understanding has led to the introduction of the term powerpenia, referring to the age-related loss of skeletal muscle power across the lifespan, and underscores the importance of prioritizing power-oriented training strategies (8, 9).

The characteristics of resistance training that lead to the greatest gains in muscle power are well known and have been studied, particularly in athletic populations (10, 11). Recent advances in training methodology have focused on improving muscle power using more efficient and specific exercise prescription (12). Among others, a velocity-based training has gained attention as a practical method to monitor and individualize training load and volume in real time using barbell velocity as a performance measure (13). A particularly promising strategy is to adjust the repetitions within the training session based on a velocity loss threshold, whereby a set is terminated as soon as a predetermined drop in movement velocity is detected. It is important to note that when using velocity loss thresholds, the intent and movement velocity must be maximal (e.g., as fast as possible) (13). Such execution of exercise was found to produce highest movement velocity, thus it is preferable method when aiming to gain muscular power (14). Various velocity loss thresholds have been used in the literature, mostly ranging from 10%, 20%, 30% and up to 40% (15).

Although the benefits of a 10% velocity loss for muscle power have been demonstrated in young and athletic individuals (15), their use in untrained adult women is less studied. Furthermore, it is unclear whether such strategies offer functional benefits over traditional resistance training protocols for untrained adult women, which typically emphasize fixed repetitions (i.e., 10), tempo movement execution (i.e., concentric to eccentric ratio 1:2 s) and training to near failure within the set. Given the increasing interest in efficient and power-oriented resistance training models for untrained adult women, research is needed to determine whether a 10% velocity loss threshold can be used to optimize training outcomes in terms of muscle strength, power, and function.

In line with the presented research gaps, this study aimed to investigate the effects of a 6-week lower limb resistance training program using a 10% velocity loss threshold compared to the standard training in untrained adult women. Primary outcomes included changes in lower limb power as well as muscular endurance and dynamic stability. We hypothesized that the VBT group would achieve similar or greater improvements with a lower training volume, providing a more efficient and targeted intervention for enhancing lower-limb power in this population.

## 2 Materials and methods

### 2.1 Study design

**Figure 1:**
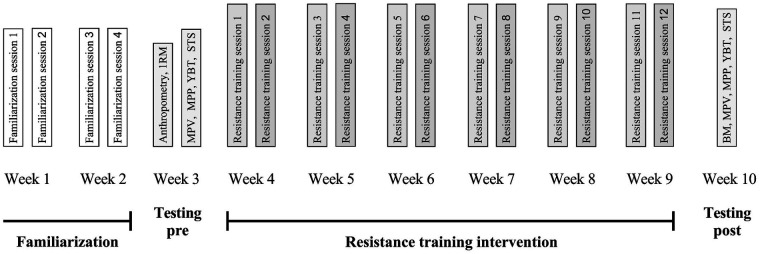
The protocol of the study including two weeks of familiarization, pre and post testing assessment, and 6-week resistance training intervention. Testing of maximum lifted weight prediction (1RM), body mass (BM), squat and deadlift mean propulsive velocity (MPV), squat and deadlift mean propulsive power (MPP), 30-seconds sit-to-stand (STS), and Y-balance test (YBT) were performed.

For the purposes of this study, a longitudinal pretest-posttest design with two groups was used. Both groups underwent resistance training with different training methods. As the participants were inexperienced in resistance training, the first two weeks were used for familiarization. In two training sessions per week (four in total), the correct execution of the exercises was learnt with no or minimal external load. In the third week, 1RM of the resistance exercises used in the training program for the lower limbs was determined. In the same week, power, muscular endurance, and function of the lower limbs were assessed using the mean propulsive velocity (MPV) and mean propulsive power (MPP) in the squat and deadlift, the 30-second sit-to-stand test (STS), and the Y-balance test (YBT), respectively. Resistance training was started in the fourth week of the protocol and was performed continuously for six weeks, twice a week, with at least 72 h between sessions. One group performed lower body exercises with a 10% velocity loss threshold (VB_10%_), while the other group performed lower body exercises with traditional 10-repetitions (STD). In the last week of the protocol, a post-test was performed to test the changes in power, muscular endurance, and dynamic stability of the lower body. The study protocol is shown in Figure 1.

### 2.2 Participants

To achieve a power of 80%, with an alpha level of 0.05, two groups, two measurements (pre and post), and a partial eta squared (*η*^2^) of 0.34 [based on the between-groups interaction effect size for hip thrust MPP reported by Montalvo-Pérez et al. (16)], we required a total sample size of 16 participants (G*Power v3.1.). The inclusion criteria were healthy adult women (between 35 and 60 years of age) with no previous experience of structured resistance training, and no musculoskeletal injuries in the previous nine months. Participants were recruited through invitations in the community near the gym where the study was conducted, resulting in a network sample. Once twenty participants had volunteered and recruitment was completed, participants were randomly allocated to either the STD or VB_10%_ group. Randomization was performed using a simple computer-generated random sequence created by a researcher who was not involved in conducting the training sessions or outcome assessments. Because of the practical nature of the intervention, neither participants nor trainers could be blinded to group assignment. The descriptive statistics of the participants can be found in Table 1. Prior to the intervention, participants were informed about the procedures and gave informed consent. The methods and interventions were reviewed and approved by the University of Primorska's Commission for Ethics in Human Subjects Research (approval number: 4264-19-6/23).

**Table 1:** Descriptive statistics and calculated one repetition maximum of lower body exercises of participants in standard resistance training group (STD) and 10% velocity loss resistance training group (VB_10%_) before the intervention.

Characteristic	STD	VB_10%_
Age	46.70 ± 6.40	44.50 ± 4.90
Body mass	66.87 ± 12.97 kg	67. 70 ± 14.36 kg
Body height	168.00 ± 4.10 cm	167.50 ± 4.10 cm
1RM squat	51.07 ± 9.07 kg	54.57 ± 12.11 kg
1RM deadlift	49.29 ± 7.10 kg	52.14 ± 10.67 kg
1RM hip thrust	51.79 ± 14.11 kg	56.43 ± 12.23 kg
1RM split squat	33.93 ± 8.95 kg	40.00 ± 11.64 kg
70% 1RM squat	35.75 ± 6.35 kg	38.20 ± 8.48 kg
70% 1RM deadlift	34.50 ± 4.97 kg	36.50 ± 7.47 kg
70% 1RM hip thrust	36.25 ± 9.88 kg	39.5 ± 8.56 kg
70% 1RM split squat	23.75 ± 6.26 kg	28.00 ± 8.15 kg

1RM, prediction of the maximum lifted weight.

### 2.3 Measurements and data collection

In the third week, the 1RM for squat, deadlift, split squat and hip thrust were calculated using an indirect method to plan the training intervention. Prior to testing, a standardized warm-up procedure was performed, which included 5 min of low-intensity stepping and dynamic stretching exercises, followed by 10 repetitions of unloaded squats, push-ups and crunches. Three minutes after the warm-up, the above exercises were performed with a submaximal load and a maximum number of repetitions that a participant could perform (17). The load and number of repetitions were used to estimate the 1RM using Brzycki's equation (18), which has been shown to be suitable for all of the above exercises (19). The results of the 1RM tests can be found in Table 1. Seventy-two hours after the tests, the primary outcome variables were tested.

#### 2.3.1 Lower body power, muscular endurance and dynamic stability

The MPV and MPP in the squat and deadlift exercises were used to determine the muscle power of the lower limbs. Prior to the tests, the standardized warm-up procedure described above was repeated. The squat was performed with a barbell placed on the upper back. During the movement, the range of motion was standardized, with the starting position at approximately 90° knee flexion. The deadlift was performed with a trapezoidal barbell and standardized with a starting position of approximately 90° hip flexion and 10° knee flexion. The external load was set at 70% 1RM, as previous studies have shown that an optimal power output is achieved at 60%–80% 1RM during lower body exercises (20). The absolute values (weight) of the 1RM for the STD and VB_10%_ groups are shown in Table 1. After a set of three repetitions for familiarization, the participants performed two sets of six consecutive repetitions at maximum lifting velocity with a 3-minute rest between sets. The linear position sensor (GymAware, Kinetic Performance Technology, Canberra, Australia) was attached to the ends of both barbells. The device was connected to an iPad (Apple Inc., Cupertino, CA, USA) via a Bluetooth connection and the manufacturer's software (GymAware Lite v2.9, GymAware, Kinetic Performance Technology, Canberra, Australia) was used to measure the MPV and MPP of all repetitions. The device has been shown to provide reliable MPP and MPV measurements during the squat at loads ranging from 40% to 90% 1RM, with intraclass correlation coefficients > 0.75 and standard errors of measurement < 8% (21). The repetition with the fastest MPV was used for further analyses. In addition, the fastest MPV of the hip thrust and split squat were assessed for the purposes of the training intervention.

Lower body muscular endurance was assessed through the 30 s STS, which was shown to be a valid tool for assessment of lower body functional capacity (21). The test procedure was demonstrated and explained prior to execution. Participants were instructed to stand up as many times as possible for 30 s, fully extend the knees and sit back down with both extremities after a verbal cue from the experimenter. A 10-second trial period was allowed before the test to familiarize themselves with the test. The standard starting position for the test was a seated position in the centre of the 45 cm chair positioned against the wall, with the arms over the shoulders and the feet on the floor. During the test, the number of complete cycles (standing up and sitting down) was counted and the time monitored with a stopwatch. If more than half of a cycle was completed in the last repetition, it was counted as complete. Two sets of STS were performed, with one minute of test time in between. The test trial with the most cycles was used for the analysis.

The YBT is considered a measure of dynamic neuromuscular control of the lower body and showed high correlation with lower limb strength in adult wom4en (22, 23). The outcome of the test depends on dynamic stability and lower limb strength, which is why it was considered a measure of lower limb functionality in this study. The YBT was performed according to the protocol described by Plisky et al. (24). Participants reached with the foot in the anterior, posteromedial and posterolateral directions while standing on each foot on a standardized device. Trunk movement was allowed while the arms were positioned akimbo. Two trials were performed on each leg. The distance pushed in each direction was measured. The recorded test result was calculated as the average distance in the anterior, posteromedial and posterolateral directions of the left and right leg.

#### 2.3.2 Resistance training programs

Two training sessions per week were carried out over a period of 6 weeks. All sessions were supervised by experienced trainer. A rest of at least 72 h was taken between the training sessions. Both training sessions consisted of identical warm-up procedure and 8 resistance exercises. Each individual exercise was completed when all sets were completed, after that the next exercise was performed. The exercises for the lower body were always performed at the beginning of the session. There was a one-minute rest between sets and a two-minute rest between exercises. In the first session of the week (resistance training sessions 1, 3, 5, 7, 9 and 11 on Figure 1), the squat (a), split squat (b), lying back extension, military press, standing rowing with elastic band, push-ups and plank were performed, while in the second session (resistance training sessions 2, 4, 6, 8, 10 and 12 on Figure 1), the hexagonal barbell deadlift (c), hip thrust (d), seated pull with elastic band, triceps bench dips, biceps curl, lateral plank and military push-ups were performed. The training programs of both groups were identical in terms of exercise selection and load. However, the number of repetitions and the lifting velocity for exercises a, b, c, and d differed between VB_10%_ and STD. The volume was calculated in kilograms for the lower limb exercises, using the Equation 1, as suggested by Haff (25). The training variables and are described in Table 2. Only individuals who attended more than 80% of the training sessions were included in the analyses.

Volume=numberofsets×numberofrepetitions×(%1RM×1RM)

**Table 2:** Training variables of resistance program in standard resistance training group (STD) and 10% velocity loss resistance training group (VB_10__%_).

Group & exercise		Training variable	Week						Volume (kg)
			1	2	3	4	5	6	
STD	Squat	Reps	10	10	10	10	10	10	6408
	Deadlift	Reps	10	10	10	10	10	10	5876
	Split squat	Reps	10	10	10	10	10	10	4201
	Hip thrust	Reps	10	10	10	10	10	10	6128
	Sets		3	3	3	3	3	3	
	%1RM		70	70	70	75	75	80	
	Total volume (kg)		22612						
VB_10%_	Squat	Reps	7.17	7.13	7.57	7.57	7.71	7.40	5086
	Deadlift	Reps	7.13	7.47	7.60	6.85	7.57	6.73	4693
	Split squat	Reps	6.77	6.73	7.30	7.13	8.00	7.00	3313
	Hip thrust	Reps	7.13	7.37	7.60	6.89	7.56	7.27	5101
	Sets		3	3	3	3	3	3	
	%1RM		70	70	70	75	75	80	
	Total volume (kg)		18193						

%1RM, percentage of prediction of the maximum lifted weight.

For VB_10%_, a set was terminated at a 10% velocity loss threshold determined using the GymAware device attached to the barbell. During lifting, real-time feedback was provided to the trainer via the iPad and the manufacturer's software. If the lifting velocity was 10% lower than the target (measured MPV during pre-test, see Figure 1), the trainer aborted the set. All other resistance exercises for the upper body and trunk were performed with the same number of sets, fixed 10 repetitions per set and a subjectively determined intensity aimed at 70, 75 or 80% 1RM. All participants except one (92%) attended all training sessions.

In the STD group, the lower body exercises were performed with the same relative load as VB_10%_, but at a standard tempo that included one second of controlled concentric and two seconds of eccentric contraction, and with a fixed 10 repetitions per set. In the same way resistance exercises Seven participants attended all training sessions, two attended 92%, and one attended 83% of the sessions.

### 2.4 Statistical analysis

Data are presented as mean and standard deviation (SD) unless otherwise stated. Prior to the analyzes, the normal distribution of the outcome variables was tested with the Shapiro–Wilk test, while homogeneity was tested with the Levene test. The unpaired samples *t*-test was used to assess the differences between STD vs. VB_10%_ in outcome variables pre-intervention and total training volume post-intervention. The effects of time (pre- vs. post-intervention), group (STD vs. VB10%), and their interaction on outcomes were examined with a 2 × 2 factorial analysis of variance (ANOVA). If the assumptions of sphericity were violated, a Greenhouse-Geisser adjustment of the *p*-values were performed. An *η*^2^ was used to assess the effect size, which was categorized as small (0.01–0.05), medium (0.06–0.14) or large (> 0.15) (26). A Holm-Bonferroni probability *post-hoc* adjustment was used to detect differences between specific time points and groups. Hedges' g effect size (ES) of differences for 2 × 2 ANOVA *post-hoc* tests was calculated and interpreted as trivial (< 0.20), small (0.20–0.49), moderate (0.50–0.79), and large (> 0.80) (26). Following significant ANOVA effects, the Holm–Bonferroni correction was applied to all *post-hoc* pairwise comparisons to control for multiple testing. The differences in the average number of repetitions per set and training session between STD and VB_10%_ were determined using a one-sample *t*-test, with a fixed 10 repetitions used for the STD group. Finally, Statistical analyzes were performed using SPSS software (version 29.0; SPSS Inc, Chicago, IL). The significance level was set at *p* < 0.05.

## 3 Results

All outcome variables showed a normal distribution (*p* > 0.10). The descriptive statistics and the results of the 2 × 2 factorial ANOVA are shown in Table 3. No differences were found between STD and VB_10%_ in pre-intervention BM (*p* = 0.893), MPV and MPP of squat and deadlift (*p* ≥ 0.499), YBT (*p* = 0.959), and STS (*p* = 0.537). ANOVA revealed a significant interaction effect between group and time for the MPV and MPP in the deadlift and the STS (F ≥ 7.39, *p* ≤ 0.014, *η*^2^ ≥ 0.29).

**Table 3:** Outcome variables descriptive statistics and results of analysis of variance.

Outcome	STD		VB_10%_		Two-way ANOVA								
	Pre ± SD (95% CI)	Post ± SD (95% CI)	Pre ± SD (95% CI)	Post ± SD (95% CI)	Group			Time			Interaction		
					F	*p*	*η* ^2^	F	*p*	η^2^	F	*p*	η^2^
BM	66.87 ± 12.97	67.11 ± 13.00	67.70 ± 14.36	67.70 ± 13.45	0.2	0.645	0.01	0.0	0.907	0.00	0.2	0.645	0.01
	(59.92–74.53)	(59.96–74.66)	(60.85–77.1)	(61.15–76.55)									
MPV_SQ_	0.98 ± 0.10	1.22 ± 0.15	(0.85–1.08)	(1.23–1.38)	0.4	0.506	0.03	85.2	<0.001	0.83	3.0	0.102	0.14
	(0.93–1.05)	(1.13–1.31)	(23.80–29.50)	(29.50–33.40)									
MPV_DE_	1.05 ± 0.19	1.18 ± 0.18	1.08 ± 0.17	1.36 ± 0.13	1.8	0.196	0.10	80.1	<0.001	0.82	11.5	0.003	0.39
	(0.94–1.16)	(1.08–1.28)	(0.97–1.18)	(1.27–1.43)									
MPP_SQ_	5.40 ± 1.34	6.64 ± 1.78	5.44 ± 1.11	7.42 ± 1.58	0.4	0.518	0.02	59.0	<0.001	0.77	3.1	0.095	0.15
	(4.68–6.14)	(5.68–7.65)	(4.74–6.13)	(6.45–8.29)									
MPP_DE_	5.48 ± 1.09	6.13 ± 1.20	5.91 ± 7.46	7.42 ± 2.04	1.7	0.204	0.09	44.3	<0.001	0.71	7.4	0.014	0.29
	(4.90–6.20)	(5.50–6.87)	(4.93–6.98)	(6.30–8.75)									
YBT	95.74 ± 8.59	105.24 ± 11.34	95.93 ± 7.03	100.83 ± 9.50	0.3	0.587	0.02	20.7	<0.001	0.54	2.1	0.163	0.11
	(90.68–100.46)	(98.66–111.57)	(91.54–99.89)	(95.07–106.06)									
STS	25.30 ± 4.60	34.70 ± 4.57	26.60 ± 4.65	31.30 ± 3.30	0.3	0.565	0.02	94.9	<0.001	0.84	19.5	0.004	0.37
	(22.60–28.10)	(32.10–37.30)	(23.80–29.50)	(29.50–33.40)									

BM, body mass in kg; MPV_SQ_, mean propulsive velocity in squat in m·s^−1^; MPV_DE_, mean propulsive velocity in deadlift in m·s^−1^. MPP_SQ_, mean propulsive power in squat in W·kg^−1^; MPP_DE_, mean propulsive power in deadlift in W·kg^−1^; YBT, Y-balance test composite in cm. STS, number of repetitions in 30-second sit-to-stand test; STD, standard resistance training group; VB_10%_, 10% velocity loss resistance training group; F, F-value; *p*, *p*-value; η^2^, partial eta squared.

**Figure 2:**
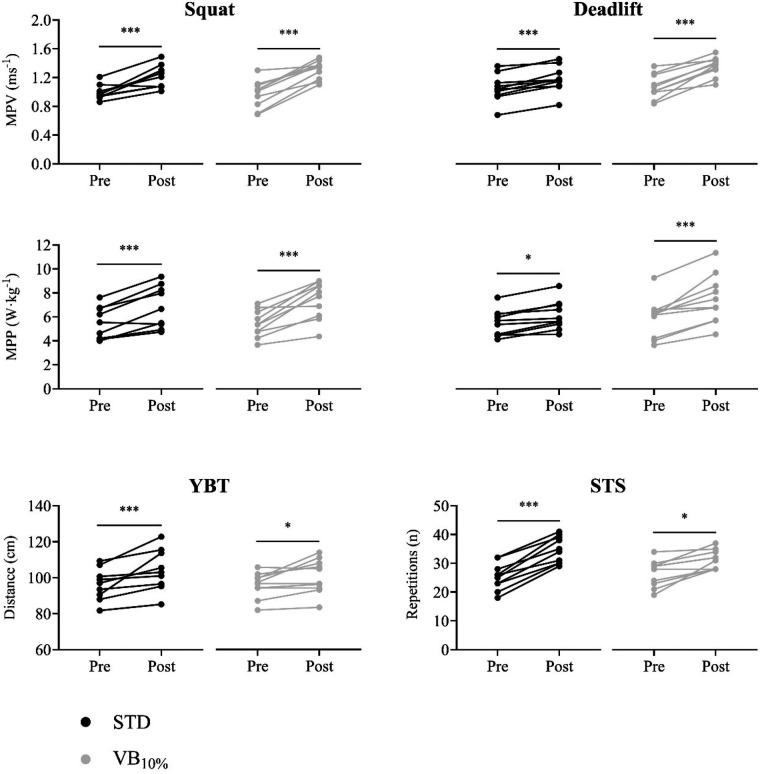
*Post-hoc* analyses showing individual pre-post differences in mean propulsive velocity (MPV) and mean propulsive power (MPP) in squat and deadlift exercises, Y-balance test (YBT), and 30-second sit-to-stand test (STS) in standard resistance training group (STD; black dots) and 10% velocity loss resistance training group (VB_10%_; grey dots). * *p* < 0.05. *** *p* < 0.001.

A significant effect of time was observed for all variables except BM (F ≥ 20.75, *p* < 0.001, *η*^2^ ≥ 0.54). As shown in the *post hoc* analyzes (Table 4 and Figure 2), the MPV and MPP of the squat and deadlift improved significantly in VB_10%_ and in STD after the training intervention (ES ≥ 1.20 and 0.83, respectively). The YBT also improved significantly after the intervention in VB_10%_ and in STD (ES = 0.80 and 1.1, respectively) as well as in STS (ES = 1.40 and 2.90, respectively).

**Table 4:** *Post-hoc* analyses showing pre-post differences in outcome variables of standard resistance training group (STD) and 10% velocity loss resistance training group (VB_10%_).

Outcome	STD pre—post			VB_10%_ pre—post		
	MD (95CI)	Δ (%)	ES	MD (95CI)	Δ (%)	ES
BM	−0.24 (−1.00–0.52)	0.4	0.22	0.00 (−0.76–0.76)	0.29	0.00
MPV_SQ_	−0.23 (−0.32 to −0.14)	23.6	1.60	−0.33 (−0.43 to −0.24)	37.5	2.20
MPV_DE_	−0.13 (−0.19 to −0.06)	12.7	0.83	−0.28 (−0.35 to −0.21)	27.7	1.20
MPP_SQ_	−1.24 (−1.78 to −0.70)	23.1	1.52	−1.98 (−2.77 to −1.20)	37.4	1.67
MPP_DE_	−0.66 (−0.94 to −0.47)	12.3	1.50	−1.56 (−2.25 to −0.86)	27.6	1.49
YBT	−11.44 (−17.28 to −5.6)	10.1	1.10	−4.59 (−10.43–1.26)	5.1	0.80
STS	−9.40 (−11.55 to −7.25)	38.9	2.90	−4.70 (−6.85 to −2.55)	19.9	1.40

BM, body mass in kg; MPV_SQ_, mean propulsive velocity in squat in m·s^−1^; MPV_DE_, mean propulsive velocity in deadlift in m·s^−1^; MPP_SQ_, mean propulsive power in squat in W·kg^−1^; MPP_DE_, mean propulsive power in deadlift in W·kg^−1^; YBT, Y-balance test composite in cm; STS, number of repetitions in 30-second sit-to-stand test; STD, standard resistance training group; VB_10%_, 10% velocity loss resistance training group; MD, mean difference; 95CI, 95% confidence interval; Δ %, relative change; ES, Hedges'g effect size; W, Watts; kg, kilograms; s, seconds; *n*, number of repetitions.

**Figure 3:**
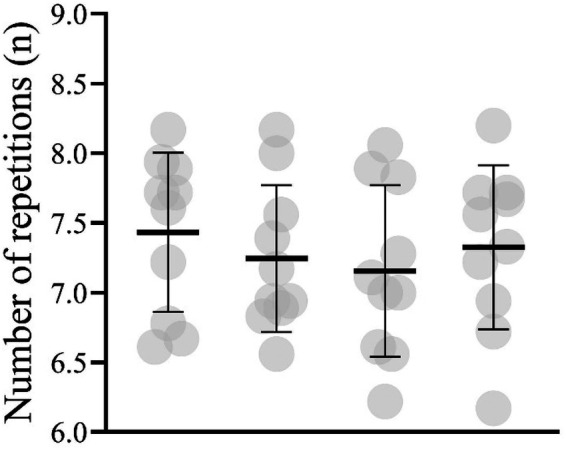
Mean (black horizontal line) and individual's (grey circles) average number of squats, deadlifts, hip thrusts, and split squats performed per set per session in 10% velocity loss resistance training group.

The number of repetitions per set and session for the lower body resistance exercises performed in the VB_10%_ are shown in Figure 3. A one-sample *t*-test showed that significantly fewer than 10 squats (7.43 ± 0.57; *p* < 0.001), deadlifts (7.25 ± 0.52; *p* < 0.001), split squats (7.12 ± 0.61; *p* < 0.001), and hip thrusts (7.33 ± 0.19; *p* < 0.001) were performed in the VB_10%_. Furthermore, total training volume for four lower limb exercises was significantly lower in the VB_10%_ group (18,193.2 ± 3,597.0 kg) as compared to STD (22,612.1 ± 2,627.7 kg), *t*(18) = 3.14, *p* = 0.006.

## 4 Discussion

The aim of this study was to compare the effectiveness of a 10% velocity loss protocol and a standard resistance training method over a 6-week period on lower limb muscular endurance, power and dynamic stability in untrained adult women. Both training strategies resulted in significant improvements in all outcomes, suggesting that biweekly resistance training, regardless of execution method, is effective in mitigating the age-related decline in muscle performance in this population. The standard training method appeared to be more beneficial for improving lower limb muscle strength and endurance, which was reflected in greater gains in STS. In contrast, the VB_10%_ approach resulted in superior improvements in MPV and MPP, indicating its potential as a more effective strategy for improving lower limb muscle power. Furthermore, the VB_10%_ group achieved these improvements with a significantly lower training volume, highlighting the practicality and efficiency of this approach for addressing early declines in lower-limb power in untrained adult women.

This is consistent with previous research in predominantly resistance-trained men showing that low thresholds of velocity loss are preferable for optimizing the development of muscle power (27–30). The results of a recent meta-analysis by Jukic et al. (15) support the use of 10%–20% velocity loss when aiming to improve vertical jump and sport-specific performance. Furthermore, the authors found that resistance training with a low velocity loss threshold (i.e., 10%) was most optimal for improving MPV at low loads (lifting velocity > 1 m·s^−1^, note that in our study a similar MPV was considered in the squat and deadlift as a measure of lower limb power). They point out that the results cannot be generalized to the female population as only one study was conducted in young women (31).

Our findings add novel evidence showing that a VB_10%_ can elicit greater improvements in lower-limb power compared with STD in untrained adult women. These observations align with recent work in elite athletes demonstrating that resistance training performed with higher movement speed and maximal intent produces superior gains in muscular power, even when external load and training volume are matched (11, 14). This literature supports our interpretation that the VB_10%_ group, who executed all repetitions with the highest movement velocity possible, experienced greater improvements in MPV and MPP despite completing fewer total repetitions. From a neuromuscular perspective, high-velocity, maximal-intent contractions have been associated with increases in muscle pennation angle (32), enhanced neural adaptations such as greater motor units recruitment and faster frequency discharge (9), and attenuation of the fast-twitch muscle fibers atrophy and number (33, 34). These functional and structural adaptations may enhance the capacity to generate force rapidly and efficiently at high contraction velocities, which could help explain the superior power gains observed in the VB_10%_ group. It is important to acknowledge that these explanations remain speculative in the context of the present study, as neuromuscular or architectural variables were not directly measured. Therefore, future research is warranted to examine the neuromuscular and structural adaptations underlying velocity-based resistance training in adult untrained women.

Together with maximal intent during every repetition, a set termination was based on a 10% velocity-loss threshold in VB_10%_. A velocity-loss termination predominantly serves to limit fatigue (35) and reduce low-quality repetitions performed under substantial velocity decline (36). Because our design did not manipulate these factors independently, we cannot fully disentangle their individual contributions. Future studies should employ factorial designs to isolate the specific effects of intent vs. velocity-loss thresholds.

In contrast to our expectations the gains in lower limb muscular strength assessed through STS were greater in STD group. This is surprising as previous studies have shown U-shaped relationship between different velocity loss thresholds and strength gains with low to moderate velocity loss shown as the most effective (15). However, STS could be discounted as a true indicator of muscle strength, although it has been reported as such in several studies (37). Strength is defined as the maximum force that a muscle or muscle group can generate at a given velocity (10), but in STS the goal is to perform as many repetitions as possible within a fixed time window against a relatively light external load (e.g., body mass). So it could be that STS performance is actually more dependent on muscle endurance. Speaking of which, previous studies have shown that a higher volume of resistance training has the greatest effect on muscular endurance (38, 39). Because the STD performed a significantly higher number of repetitions of the lower body exercises, the gains in STS were more pronounced than in VB_10%_.

It should be noted that both groups improved YBT without a significant interaction effect. The reason for this could be that dynamic neuromuscular control of the lower body depends on many factors, including muscle-specific factors related to increasing muscle strength, power or endurance. As these were improved in the STD and also in the VB_10%_, the improvements in lower body function were similar between the two groups. However, it should be noted that the VB_10%_ participants performed 27% fewer repetitions of the lower limb exercises than the STD participants (∼524 and ∼715 repetitions in total and a total volume of 22,612 and 18,193 kg, respectively). This is a common observation when using low velocity loss thresholds for the purpose of resistance training, even in the older adult population (40). The use of VB_10%_ in adult women could therefore be a more efficient modality of resistance training when aiming to improve lower limb muscle power, endurance, and dynamic stability.

The results of this study provide valuable practical guidance for the design of resistance training programs for untrained adult women. While STD-like training methods remain effective for improving overall muscle power, endurance, and lower limb function, velocity-based training, particularly a 10% velocity loss threshold, may offer a more efficient and targeted approach to improving muscle power. Given the challenges of dynapenia and powerpenia that occur as women age, incorporating 10% velocity loss protocols may help to both improve muscle power and minimize fatigue and training time. Such an approach may be particularly beneficial for women with limited training history or time availability and may promote greater adherence and long-term commitment to resistance training. Therefore, exercise professionals and practitioners working with adult women should consider combining traditional and velocity-based methods to optimize muscle function, compensate for age-related decline, and promote functional independence.

Finally, several limitations of this study should be considered when interpreting the results. First, the relatively small sample size and short, 6-week intervention duration may limit the generalizability of the findings, particularly regarding longer-term neuromuscular adaptations. Second, the study did not include a double-blind design, which may introduce expectancy bias in participants or trainers. Third, neither dietary intake nor physical activity outside the intervention was monitored or controlled, which could have influenced the outcomes. Additionally, although improvements in muscle power and function were observed, underlying neural adaptations, muscle mechanics, or changes in muscle architecture were not directly assessed. As the study included only untrained adult women, caution should be exercised when extrapolating the findings to older populations or individuals with different training backgrounds.

## 5 Conclusion

This study showed that both traditional and velocity-based resistance training effectively improved lower limb muscle power, muscular endurance, and dynamic stability in untrained adult women over a 6-week period. While the standard protocol was associated with greater gains in lower limb muscular endurance, the 10% velocity loss threshold resulted in superior improvements in muscular power despite requiring significantly fewer repetitions. These results suggest that velocity-loss strategies offer more efficient and targeted alternative for improving muscle function, particularly lower-limb power, which is often not adequately addressed in standard resistance training programs for untrained adult women. Incorporating velocity-based resistance training into exercise may therefore increase efficiency and should be considered a valuable strategy for maintaining muscular capacity and function across the adult lifespan of woman.

## Data Availability

The data analyzed in this study is subject to the following licenses/restrictions: as the data that support the findings of this study contain information that could compromise the privacy of research participants they are available on request from the author (MS). Requests to access these datasets should be directed to matic.sasek@fvz.upr.si.
